# Toward Advanced Sensing and Data-Driven Approaches for Maturity Assessment of Indeterminate Peanut Cropping Systems: Review of Current State and Prospects

**DOI:** 10.3390/s26072208

**Published:** 2026-04-02

**Authors:** Sathish Raymond Emmanuel Sahayaraj, Abhilash K. Chandel, Pius Jjagwe, Ranadheer Reddy Vennam, Maria Balota, Arunachalam Manimozhian

**Affiliations:** 1 Department of Biological Systems Engineering, Virginia Tech, Blacksburg, VA 24061, USA; sathish777@vt.edu (S.R.E.S.); pjjagwe@vt.edu (P.J.); arunam@vt.edu (A.M.); 2 Tidewater Agricultural Research and Extension Center, Virginia Tech, Suffolk, VA 23437, USA; ranadheerv@vt.edu (R.R.V.); mbalota@vt.edu (M.B.); 3 School of Plant and Environmental Sciences, Virginia Tech, Blacksburg, VA 24061, USA

**Keywords:** peanut maturity, traditional methods, high-throughput methods, artificial intelligence, end-to-end solution, precision harvest and crop management

## Abstract

**Highlights:**

**What are the main findings?**

- Peanut maturity assessment is inherently difficult because pods develop below ground and mature asynchronously, making traditional methods (e.g., hull scrape and pod blasting) destructive, labor-intensive, and spatially limited. This review provides the focused synthesis of traditional, sensing-based, and AI-supported approaches for peanut maturity assessment.
- Emerging non-invasive sensing technologies combined with AI and geospatial analytics show strong potential for scalable maturity prediction, but current approaches remain limited by the insufficient integration of agroclimatic, phenological, and cultivar-specific factors.

**What are the implications of the main findings?**

- There is a critical need to transition toward integrated, data-driven maturity assessment frameworks that combine multimodal sensing, AI modeling, and spatial analytics to enable accurate and scalable harvest decision-making.
- Developing robust ground-truth datasets and user-oriented digital decision-support systems will be essential to translate sensing and modeling advances into practical tools that improve harvest timing, productivity, and economic returns for peanut growers.

**Abstract:**

Determining the optimal harvest time is among the most critical economic decisions for peanut (*Arachis hypogaea* L.) growers, directly influencing yield, quality, and market value. Unlike many other crops, peanuts are indeterminate, continuing to flower and produce pods throughout their life cycle. As a result, pod development and maturation are asynchronous, making harvest timing particularly challenging. Conventional maturity estimation techniques, including the hull scrape method, pod blasting, and visual maturity profiling, are invasive, labor-intensive, time-consuming, and spatially limited. Moreover, differences in cultivar maturity rates and agroclimatic conditions exacerbate inconsistencies in maturity prediction. These challenges highlight the urgent need for scalable, objective, and data-driven methods to support growers in achieving optimal harvest outcomes. This review synthesizes the current understanding of peanut pod maturity and evaluates existing traditional and non-invasive approaches for maturity estimation. It aims to identify the limitations of conventional techniques and explore the integration of advanced sensing technologies, artificial intelligence (AI), and geospatial analytics to enhance precision and scalability in peanut maturity assessment and harvest decision-making. This review examines traditional destructive techniques such as the hull scrape method and pod blasting, followed by emerging non-invasive methods employing proximal and remote sensing platforms. Applications of vegetation indices, multispectral and hyperspectral imaging, and AI-based data analytics are discussed in the context of maturity prediction. Additionally, the potential of multimodal remote sensing data fusion and digital frameworks integrating spatial big data analytics, centralized data management, and cloud-based graphical interfaces is explored as a pathway toward end-to-end decision-support systems. Recent advances in non-invasive sensing and AI-assisted modeling have demonstrated significant improvements in scalability, precision, and automation compared with traditional manual approaches. However, their effectiveness remains constrained by the limited inclusion of agroclimatic, phenological, and cultivar-specific variables. Furthermore, the translation of model outputs into actionable, field-level harvest decisions is still underdeveloped, underscoring the need for integrated, user-centric digital infrastructure. Achieving a robust and transferable digital peanut maturity estimation system will require comprehensive ground-truth data across cultivars, regions, and growing seasons. Multidisciplinary collaborations among agronomists, data scientists, growers, and technology providers will be essential for developing practical, field-ready solutions. Integrating AI, multimodal sensing, and geospatial analytics holds immense potential to transform peanut maturity estimation. Such innovations promise to enhance harvest precision, economic returns, and sustainability while reducing manual effort and uncertainty, ultimately improving the efficiency and quality of life for peanut producers worldwide.

## 1. Introduction

Peanuts, also known as groundnuts, are a leguminous crop domesticated in South America [1]. Its binomial name was derived from the Greek words “*Arachis*”, which denotes its classification as a legume, and “*hypogaea*”, which describes its unique geocarpic nature, where pods develop and mature underground [2,3]. As a globally significant oilseed legume, peanuts are cultivated in over 100 countries. Global production exceeded 50 million metric tons in 2024, with China (38%), India (14%), Nigeria (9%), the United States (6%), and Burma (3%) collectively contributing to about 70% of the total global production [4]. Peanuts are one of the world’s most important crops in terms of economic and nutritional value. They serve as a major source of oil and edible seeds and play a vital role in food security for developing and underdeveloped countries [5]. One of the major agronomic challenges in peanut production is the underground pod development, making it difficult to assess or estimate the status of pod development and maturity. Peanut crops are indeterminate in nature and continue to grow, flower, and produce pods below ground as long as agroclimatic conditions remain favorable. As a result, plants do not achieve uniform pod maturity at any single point in time, i.e., all the pods are unable to mature at the same time. Therefore, it is highly critical to identify when the peanut pods will attain optimum maturity to time precision harvest for maximum yield and quality.

Maturity of peanuts directly impacts their nutritional and oil content values, flavor, and, therefore, market value [6]. Therefore, untimely harvests may result in pre-mature or over-matured kernels where pre-matured kernels retain relatively higher moisture and require artificial drying to meet market standards (~18% moisture) or else discounted prices are offered at the sales point. Artificial drying is mostly performed using propane-run dryers and therefore turns out to be expensive. Pre-mature peanuts are susceptible to post-harvest fungal infection, particularly by Aspergillus flavus and Aspergillus parasiticus, which can lead to aflatoxin contamination under conditions of high temperature, elevated kernel moisture, mechanical damage, and improper drying or storage, posing serious risk to human health and food safety [7,8]. Immature seeds also suffer from poor germination and growth over generations and are unable to capture and capitalize on resources and attain optimum yield in the future [6]. Conversely, over-matured peanuts experience weakened pegs and therefore detach even with minimal disturbance. Detached pegs are therefore retained in the soil during harvest, leading to non-harvestable crop loss. Additionally, over-mature pods are also vulnerable to aflatoxin contamination, particularly under hot soil conditions, low soil moisture, and when pods shells are cracked or damaged during field exposure, making timely harvest a critical risk-mitigation strategy in dryland production systems [7]. In practice, peanuts are typically harvested when approximately 70% of the pods per plant are estimated to have attained maturity [9]; however, this threshold still leaves a significant proportion of immature pods in the field during harvest [10,11]. Previous studies have shown that digging as little as seven days before optimum maturity can reduce net returns by $100–150 per acre under current market conditions [12]. Yield losses from pre-mature harvesting are commonly associated with kernel shrinkage, reduced seed filling, and a higher proportion of low-grade kernels that are diverted to oil processing rather than the edible market, resulting in both quantitative and qualitative penalties [11,12]. Therefore, harvests at both pre-mature and over-mature stages lead to significant loss in yield as well as quality attributes such as phenolic compound, antioxidant, carbohydrate, protein, lipid, and oil content [13,14,15].

The existing peanut maturity estimation methods are predominantly manual and are limited by low throughput, labor-intensiveness, subjectivity, and costs, for example, the widely used hull scrape method and pod blasting with color-based maturity profiling [10]. Such methods are limited in quantifying spatial field variations in maturity and therefore do not provide a true picture of field-level maturity to initiate precision harvesting operations. To overcome these limitations, researchers are exploring non-invasive and high-throughput methods such as remote sensing, machine learning (ML), artificial intelligence (AI) models, and sensor-based techniques to determine peanut maturity, which tend to be non-subjective and offer a geospatial and scalable solution to peanut growers [16,17,18]. Given the advent of technology in agricultural production management and increasing market competitiveness, adopting digital solutions to enhance throughput capacity and efficiency in peanut production is the best possible way forward. Therefore, the major goal of this article is to provide a comprehensive understanding of the traditional, emerging, and potentially novel methods driven by artificial intelligence for estimating peanut maturity necessitated by the challenges of indeterminate peanut cropping systems. This article also synthesizes the existing methods for maturity estimation, highlighting their effectiveness, advantages, and limitations. The scientific databases such as Science Direct, Scopus, IEEE, and Web of Science have been used to examine evolving research over the years for comprehensively estimating peanut maturity. This review is developed with the capacity to answer the following questions of the readers as they reach the end of this article.

(1) Why is peanut maturity estimation critical for optimizing harvest outcomes and which key environmental and management factors influence maturity in indeterminate peanut crops?
(2) What traditional, field- and laboratory-based (often destructive) methods are used for peanut maturity estimation and what are their major challenges and limitations?
(3) What emerging, non-invasive and high-throughput phenotyping technologies have been explored for peanut maturity estimation?
(4) What does the existing literature suggests about the potential and practical consideration of integrating remote sensing, AI and digital decision-support frameworks for accurate, scalable, and field-level peanut maturity and harvest management for peanut producers?

## 2. Review Methodology

**Figure 1:**
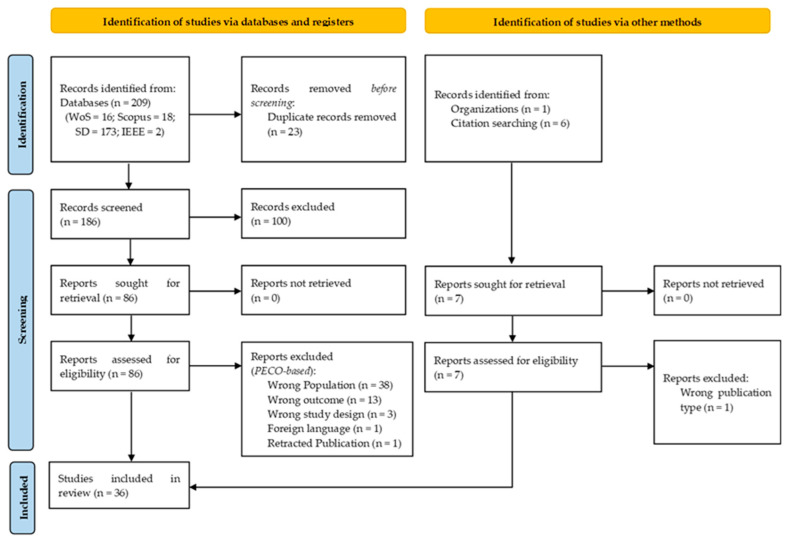
The PRISMA-style flow diagram illustrating the identification, screening, eligibility assessment, and final inclusion of studies for the systematic review on peanut maturity estimation.

This review was conducted following a PRISMA-style systematic review approach to ensure transparency, consistency, and reproducibility in identifying, screening, and synthesizing the literature related to peanut maturity estimation. A predefined search strategy, explicit inclusion and exclusion criteria, and a staged screening process were applied to systematically evaluate the available evidence [19]. The review workflow comprised the sequential phases of study identification, title and abstract screening, full-text eligibility assessment, and final inclusion. The overall selection process and record flow are summarized using a PRISMA-style diagram (Figure 1). While the foundational literature on peanut physiology and traditional maturity assessment methods was consulted to provide the background, the studies were not included unless they satisfied all inclusion criteria. These studies described pod development, kernel characteristics, and maturity indices that growers have traditionally used to decide harvest timing. Methods such as pod blasting and calculations of growing degree days (GDDs) were considered essential references, since they remain widely applied in maturity assessment and continue to guide field practices. Only studies meeting the predefined eligibility criteria were included in the PRISMA-based systematic synthesis.

### 2.1. Literature Identification and Search Strategy

The relevant literature was identified through a structured search of major (i) bibliographic databases covering agricultural sciences, remote sensing, and computational analytics and (ii) other methods (Figure 1). The databases searched included Scopus, Web of Science Core Collection, ScienceDirect, and IEEE Xplore. The literature search was conducted on 28 January 2026 and covered studies published between 1993 and 2025. Searches were limited to peer-reviewed journal articles. A keyword-based search strategy was developed to capture the literature addressing peanut maturity estimation using traditional, sensing-based, and data-driven approaches. Search terms were grouped into four conceptual categories: (i) crop terms (“peanut”, “groundnut”), (ii) maturity terms (“maturity”, “pod maturity”, “harvest maturity”), (iii) sensing and phenotyping terms (“remote sensing”, “proximal sensing”, “phenotyping”, “canopy reflectance”, “vegetation index”, “multispectral”, “hyperspectral”, “thermal”), and (iv) analytical methods (“machine learning”, “artificial intelligence”, “regression”, “classification”). Boolean operators were applied within and across groups to balance coverage and specificity. Database-specific constraints (e.g., Boolean connector limits) were respected. The final search strings were applied consistently across all databases during the identification phase. Two distinct SQL-style representative search strings were applied across databases to illustrate the Boolean structure used in this review. Query 1 was used for Scopus, Web of Science, and IEEE Xplore, while Query 2 (a shortened version) was used for ScienceDirect due to the eight Boolean-connector limit per field. In total, 209 records were identified across databases: Scopus (n = 18), Web of Science (n = 16), ScienceDirect (n = 173), and IEEE Xplore (n = 2). All records were exported in the RIS format and imported into Rayyan (https://new.rayyan.ai/ (accessed on 11 September 2025)), for reference management and screening.
-- Search String 1: For Scopus, Web of Science, IEEE Xplore (“peanut” OR “groundnut” OR “Arachis hypogaea”) AND (“maturity” OR “pod maturity” OR “harvest maturity” OR “maturity index” OR “PMI”) AND (“phenotyping” OR “canopy reflectance” OR “spectral signature” OR “vegetation index” OR “hyperspectral” OR “multispectral” OR “thermal” OR “proximal sensing” OR “remote sensing”) AND (“machine learning” OR “artificial intelligence” OR “neural network” OR “deep learning” OR “regression model” OR “classification”)  -- Search String 2: For ScienceDirect (shortened due to 8-connector limit) (“peanut” OR “groundnut”) AND (“maturity” OR “pod maturity” OR “harvest maturity”) AND (“remote sensing” OR “proximal sensing” OR “canopy reflectance”) AND (“machine learning” OR “artificial intelligence”)

### 2.2. Inclusion and Exclusion Criteria

Studies were included if they focused on peanuts (*Arachis hypogaea* L.) and addressed maturity estimation using remote sensing, proximal sensing, or quantitative modeling approaches. In addition to studies explicitly targeting maturity, the research examining closely related proxy outcomes such as yield, grade, chlorophyll content, or biomass was retained only when the analytical framework, vegetation indices (VIs), or modeling strategy were directly transferable to peanut maturity assessment. Studies were excluded if they were background or conceptual articles used only for contextual framing, focused on non-peanut crops (wrong population), addressed outcomes not transferable to maturity estimation (wrong outcome), were not original peer-reviewed research (e.g., reviews, editorials, conference abstracts; wrong publication type), employed study designs unsuitable for quantitative maturity assessment (e.g., surveys, economic or policy studies; wrong study design), or were published in a foreign language without an accessible English full text. The inclusion and exclusion criteria were defined using a PECO-based logic (population, exposure, comparison, outcome), where the population was peanut, the exposure comprised remote sensing, proximal sensing, or quantitative modeling approaches, comparisons involved different sensing platforms, data sources, or analytical methods, and the outcome focused on maturity estimation or directly transferable proxy indicators [20].

### 2.3. Title and Abstract Screening

Screening was conducted in two sequential stages using Rayyan. Title/abstract screening and full-text eligibility assessment were performed independently by three reviewers and then consolidated through discussion. No major disagreements arose during study selection. First, titles and abstracts of the 186 unique records were screened against the predefined PECO-informed eligibility criteria. During this stage, 100 records were excluded, primarily because they were background or conceptual articles, wrong publication types (e.g., reviews, editorials, or conference abstracts), or were otherwise outside the review scope based on title and abstract. The remaining 86 records were sought for retrieval and advanced to full-text assessment. A conservative approach was applied during title and abstract screening, whereby records with ambiguous relevance were retained for full-text review to minimize the risk of erroneous exclusion.

### 2.4. Full-Text Eligibility Assessment

Full-text articles were evaluated to confirm relevance, methodological transparency, and alignment with the review objectives using PECO-informed eligibility criteria. Of the 86 articles assessed in full, 56 studies were excluded because they focused on non-peanut crops (wrong population; n = 38), examined outcomes not directly transferable to maturity estimation (wrong outcome; n = 13), employed study designs unsuitable for quantitative maturity assessment (wrong study design; n = 3), were published in a foreign language without accessible English full text (n = 1), or had been formally retracted (n = 1). The remaining 30 studies met all eligibility requirements and were retained for inclusion in the systematic synthesis.

### 2.5. Final Study Inclusion

In addition to database searching, 7 research articles relevant to peanut maturity estimation were identified through other methods, including organizational repositories (n = 1) such as extension or research program publications maintained by agricultural institutions and citation searching (n = 6), where relevant studies were identified by examining the reference lists of key peer-reviewed articles. These studies provided foundational or complementary evidence on peanut maturity assessment but were not captured by the predefined database search strings. All records identified through other methods were managed using Rayyan and were advanced directly to full-text eligibility assessment. Overall, 1 report was excluded due to wrong publication type, resulting in 6 studies retained from other methods. Together with studies identified through database searching, a total of 36 studies were identified and included in the final systematic review (Figure 1).

The included studies form the primary evidence base for the synthesis presented in subsequent sections. Data extraction was conducted using the predefined criteria to record study characteristics such as sensing modality, platform, analytical/modeling approach, and reported outcomes relevant to peanut maturity assessment. A formal risk-of-bias or critical appraisal was not conducted because the included studies were methodologically heterogeneous and mainly comprised sensing- and AI/ML-based designs. However, limitations related to cultivar coverage, site specificity, seasonal scope, and sample size were explicitly considered during evidence synthesis and interpretation. The foundational literature on peanut physiology, agroclimatic influences, and traditional maturity assessment methods is incorporated to provide the essential context and to support the interpretation of sensing- and AI-based approaches. Section 3, Section 4 and Section 5 therefore establish the agronomic and practice-oriented background, while Section 6 and Section 7 synthesize the systematically selected studies to evaluate advancements in sensing technologies, artificial intelligence, and digital maturity assessment frameworks.

## 3. Peanut Crop Physiology and Phenological Stages

**Figure 2:**
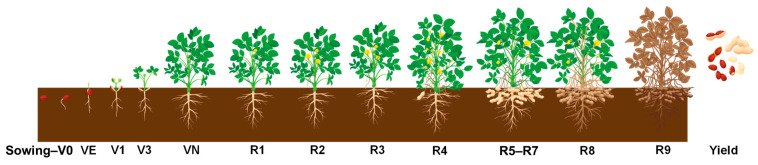
Peanut growth stages (V0—zero-leaf stage; post-emergence; V1—first tetrafoliate stage; V3—third tetrafoliate stage; VN—nth tetrafoliate leaf stage; R1—beginning bloom; R2—beginning peg; R3—beginning pod; R4—full pod; R5–R7—beginning seed, full seed, and beginning maturity; R8—harvest maturity; R9—over-maturity).

Peanut is a member of the family Fabaceae, which includes legumes such as soybean (*Glycine max* L.) and pea (*Pisum sativum* L.). This classification establishes its relationship with other nitrogen-fixing crops, which contribute significantly to soil health [21]. Peanut seeds are dicotyledonous, containing two cotyledons that serve as the first true leaves and store food reserves for young seedlings during germination and early development [9]. Each seed includes the dormant shoot (plumule) and the root (radicle) [9,22]. Under optimal soil moisture and temperature conditions, germination begins within 3–7 days, during which the radicle develops into the primary root and the hypocotyl extends to form the main stem. Lateral roots emerge within the first few days, supporting the early establishment. The cotyledons split during germination, exposing the shoot primordia that extends from the epicotyl, which contains the first true leaves and becomes the main stem. Peanut leaves are compounded with four leaflets, making them tetrafoliate, and arranged alternately along the main stem and lateral branches [22]. Depending on the variety, Virginia-type peanuts have intermediate growth habits between runner and bunch types. These plants generally grow 30–70 cm tall and form a bushy canopy [23]. Figure 2 illustrates peanut germination and its key phenological growth stages, and Table 1 describes all these growth stages with visual attributes detailed by Boote [9], Balota and Phipps [24], and Jordan [25].

Flowering begins around 30–45 days after germination and continues throughout the growing season. Flowers emerge at the leaf axils on branches and the main stem and form spike-like, simple, or compound monopodial inflorescences [22]. Each inflorescence typically has up to five flowers, though three are the most common, but at any given time, only one flower per inflorescence is open [22]. Peanuts are self-pollinated, as each flower contains both male and female reproductive structures, enabling pod development [22]. However, only 15–20% of flowers successfully develop into pods [26]. Fertilization begins when pollen germinates on the stigma, forming a pollen tube that travels through the style to the embryo sac [27]. Double fertilization produces the embryo and endosperm, initiating pod development [27]. After fertilization, a pointed stalk called “peg” emerges and grows downward into the soil. At the tip of each peg, a pod is developed that houses seeds [22,27]. Due to its indeterminate growth habit, flowering and pod development occur simultaneously throughout the season, resulting in pods of different maturity stages at different instances as well as at harvest [27]. Peanut pod development is a crucial phase that directly influences the yield, quality, and economic returns for farmers [28]. Pod formation begins from the stage R3 onwards and continues with development to full pod (R4), beginning seed (R5), and full seed (R6) stages [9]. Mature pods have greater grade characteristics and optimum oil (45–55%) and protein (25–30%) content, making them more valuable for nutrition and marketability [24,29,30].

The pods of different maturity stages are present on the same plant at the time of harvest, which poses great challenges for making harvest decisions. The influence of vegetative growth regulation on peanut development has been evaluated in multiple studies using growth regulator treatment (prohexadione calcium in the form of Apogee) and no treatment (No-Apogee) systems. Jordan et al. [31] reported that growth regulator application improved row visibility and influenced yield responses across Virginia-type cultivars, although treatment effects varied among genotypes and environments. Similarly, Gaudin et al. [32] demonstrated that prohexadione calcium application timing and rate affected vegetative growth and yield in Mississippi production systems, with responses dependent on cultivar and the management context. Collectively, these findings suggest that, while canopy growth regulation can modify plant architecture and harvest efficiency, maturity progression and final production outcomes remain strongly influenced by cultivar characteristics and environmental conditions.

## 4. Agroclimatic Factors That Impact Peanut Pod Maturity

Temperature is one of the most critical environmental factors influencing peanut development. In general, peanuts require at least 150 frost-free days after planting for successful production [33]. The ideal temperature for vegetative growth is 25–28 °C, while for reproductive stages, including flowering and pod development is 22–24 °C [34]. Nighttime temperature plays an important role in biomass accumulation. Prasad et al. [35] reported that growth ceases when the nighttime temperature falls below 14 °C in peanuts. These authors noted that, when day/night temperatures sharply fall from 32/26 °C to 17/11 °C, the duration of germination, emergence, and vegetative growth increases from 31 to 75 days. Temperatures at or exceeding 35 °C can damage the physiological and metabolic cellular processes and be detrimental to dry matter production, flower-to-peg conversion, pod numbers, and seed weight [29,36,37].

Water availability is another important abiotic factor affecting peanut production. On average, peanuts require 500–700 mm of rain during the growing season for optimum yields [35,38]. Sub-optimal soil moisture during the early season or 50 days after emergence was reported to have delayed germination and reduced leaf area and biomass accumulation [36,39]. Drought during reproductive development delays flowering by 1–2 days depending on the severity and reduces flower number, causing pollen sterility, abortion of embryo, delayed peg initiation and elongation, and pod initiation [35,39].

The third key factor after temperature and soil moisture is sunlight or incoming radiation. The photoperiod is an essential component that influences flowering and pod development. Insufficient sunlight during critical growth stages can hinder pod formation and delay pod maturity [40]. Solar radiation at longer daylight (>13 h) increases vegetative growth and the rate of crop growth and decreases the proportion of photosynthate to pods, while shorter daylight (<12 h) increases the number of flowers, peg formations, and pod development [41,42]. Such fluctuating conditions contribute to uneven pod maturity, further complicating the determination of optimal peanut digging time.

The genotype–environment (GXE) interaction is the next critical factor that influences peanut maturity, with GDDs serving as a key determinant of developmental stages across regions. Runner-type peanuts such as Georgia-09B and Tifguard typically reach maturity in 135–145 days after planting (DAP) in warmer climates like in Georgia, USA, where rapid heat unit accumulation accelerates development. In contrast, lower GDD accumulation in Virginia, USA, can delay maturity beyond 155 DAP, increasing the risk of early freeze damage [37,43,44]. The diverse environmental conditions across peanut-growing regions in the world have fostered breeding programs to develop cultivars with varied time-to-maturation traits. Early maturing peanut varieties are favored in areas with limited water supply or cool end-of-season temperatures to ensure complete pod filling and maintain yield and quality [43].

Crop management strategies also influence peanut pod maturity and their uniformity across the field; hence, for example, adjusting sowing dates to align with soil temperatures is an important planting decision considered by the growers [45]. Planting peanuts when soil temperatures at a four-inch depth reach 18 °C for three consecutive days accelerates germination and promotes even seedling emergence, reducing the variability of maturity dates in a field by up to 10–15 days compared to earlier planting in cooler soils where higher spatial variability can be expected [34,37,46]. Excessive irrigation or frequent rainfall can also delay maturity by promoting continued vegetative growth, while drought stress can slow the accumulation of GDDs [9,37]. Early planting in cooler soils and late-season nitrogen applications could promote vegetative growth and delay overall maturity [9,37].

More broadly, nutrient availability (both macro- and micronutrients) primarily influences peanut maturity indirectly by modifying vegetative growth, phenological duration, and source–sink balance, often in the interaction with temperature and soil moisture conditions [47,48]. In addition to abiotic factors, biotic factors also play an important role in affecting peanut pod maturity. Foliar diseases like leaf spot and sclerotinia blight can cause excessive defoliation and can force early harvest before optimal pod maturity is reached [49]. Planting peanuts in intercropping systems is also less favorable as those can hinder peanut pod development by intensifying competition for resources like nutrients and sunlight [50]. Previous physiological and modeling studies have demonstrated that peanut pod development and maturity progression are strongly regulated by temperature accumulation, photoperiod, and associated agroclimatic factors, including photoperiod, solar radiation, nutrient availability, and soil moisture [34,51,52]. More recent multi-year, multi-location modeling studies in mid-southern United States have demonstrated that GDD-based maturity indices can inform optimal digging time for the cultivars Georgia-06G and IPG-914. However, substantial region-, cultivar-, and season-specific variability remains, and peanut maturity is also influenced by environmental factors that are not fully captured by GDDs [53], which has implications for sensing-based and data-driven maturity estimation approaches.

## 5. Traditional Methods of Peanut Maturity Estimation

The evaluation of peanut pod maturity is essential for determining the optimal digging time to maximize yield, quality, and economic profitability for farmers. This section details conventional destructive and non-destructive methods typically used for determining peanut maturity as well as their advantages and limitations. Destructive methods involve direct physical interaction with pods, often requiring scraping, cutting, or opening to assess maturity. The comparison of all such conventional methods is presented in Table 2.

### 5.1. Shellout Method

The shellout (SO) method, also known as the interior hull color method, is one of the oldest and most destructive methods to determine peanut pod maturity [12,54]. Although it is not widely used in recent times, this method is based on the visual observation of the inner hull surface of the peanut pods after manually splitting them to differentiate between mature and immature pods. This technique is particularly effective for assessing Spanish market-type varieties, where the correlation between peanut hull color and kernel maturity is well matched [55]. The internal hull color is visually inspected or measured using color-measuring devices such as gardener automatic color and color difference meter [55]. The hull and seed colors typically transition from white to dark brown and white to dark pink as the pod matures. This color transition was attributed to the aging of vein cells near the internal hull surface and the accumulation of pigments. The darkening of veins is used as a visual indicator of pod maturity, with a study showing that the Rd scale (reflectance) can effectively quantify this process. An “Rd” value below 30 generally indicates a mature sample, with values ranging from 15.49 to 43.87 in studied samples [55]. Okada et al. [56] used internal hull color with the SO method as an evaluator of peanut maturity at different phenological stages.

The SO method has been described as a reliable method for assessing peanut pod maturity due to its strong and consistent correlations observed between peanut hull color and kernel density and light transmittance of oil over four days of curing [55]. The method’s simplicity allows for easy implementation in small-scale operations or research settings for Spanish market-type cultivars. However, this method has several notable limitations. It requires the labor-intensive process of digging up samples, followed by shelling and manually segregating both hulls and kernels based on visual maturity assessments [55]. The classification relies on the subjective evaluations of factors such as kernel size, color, shriveling, and internal hull color, which can introduce variability and reduce the consistency of maturity determination. The use of color-measuring devices can improve consistency by minimizing subjectivity, but the method remains labor-intensive and restricted in sampling accuracy to estimate maturity at the field scale.

### 5.2. Methanolic Extract Method

Developed in 1975, the methanolic extract (ME) method offered an objective approach to assessing peanut maturity based on the light transmittance measurements of pod extracts [57]. This technique involves separating freshly dug peanut pods from vines, blending them with methanol (2 mL per gram of pods), and analyzing the resulting extract using a spectrophotometer. As peanuts matured, the methanolic extract darkened, resulting in lower light transmittance values. The study reported a decrease in transmittance from 77% at 99 days after planting (DAP) to 59% at 153 DAP, followed by an increase to 67% at 167 DAP. The economic impact of harvest timing based on ME values was significant, with peanuts with optimal maturity (59% transmittance) yielding $1023 per acre, compared to under-mature ones (77% transmittance) at $234 per acre and over-mature ones (67% transmittance) at $850 per acre. These results demonstrated the ME method’s potential for identifying the ideal harvest window to maximize economic returns, providing a more quantitative approach to maturity assessment compared to manual visual methods [57]. The ME method is advantageous because it provides both objective and quantifiable data with less individual bias when compared to other methods. The method is relatively simple to perform and adaptable to field- and laboratory-based studies, and the result is repeatable. However, this method relies on collecting samples from the field, which may be required in large numbers to quantify in-field variability, and this can be highly laborious. More so, the need of setup for blending samples with methanol and equipment such as spectrophotometers to observe light transmittance can be expensive and often impractical for the growers to adopt for direct use.

### 5.3. Arginine Maturity Index Method

The arginine maturity index (AMI) method, developed by Johnson et al. [58], provides an objective and automated approach to estimate peanut maturity, specifically used in the past for Virginia-type cultivars. This method quantifies the levels of arginine, an essential amino acid in peanut kernels whose content changes during maturation. The process involves sampling peanuts at various developmental stages and analyzing the kernels to determine arginine content. A 30 g subsample of peanut kernels is homogenized for 30 s in 200 mL of 2% trichloroacetic acid to produce a filtrate, which is then processed using the Sakaguchi method, an automated colorimetric technique where arginine reacts with specific chemicals to produce a measurable color change [59].

During a three-year study conducted in North Carolina and Virginia (1975–1977), researchers evaluated four Virginia-type cultivars (NC 17, NC 5, Florigiant, and Va 56R) to explore the relationship between AMI values, peanut maturity, yield, and market-grade quality. Peanut samples were collected weekly from late August to late October at various seed development stages. Results indicated that AMI values decreased from early sampling dates (150–250) to minimum values around 70–125 as peanuts reached optimal maturity. Peanuts harvested at or near these minimum AMI values exhibited the highest yields and best market-grade quality [58,59,60]. The AMI method demonstrated its potential as an objective tool for predicting peanut maturity compared to traditional subjective methods. However, significant variations in AMI values were observed across regions and cultivars, suggesting that regional calibration may be necessary for accurate implementation. Additionally, extreme weather conditions such as freezing or snow before harvest can impact both yields and AMI values [58,61]. However, like the methanolic extract method, the AMI method is also feasible only for laboratory-based maturity evaluations and relies on acquiring a lot of field samples to quantify field representable maturity variability, and this can be highly laborious and expensive. For these reasons, the AMI method is impractical for growers’ use.

### 5.4. Seed Hull Maturity Index

The seed hull maturity index (SHMI) is a peanut maturity estimation method that uses the seed–hull ratio, which involves calculating the ratio of seed weight to hull weight, referred to as fresh maturity index (FMI) or dried maturity index (DMI), based on fresh or air-dried dry weights [62]. The method requires collecting peanut pod samples at various harvest dates, then ranking them by physiological maturity, separating seeds from hulls, measuring their weights both fresh and air-dried, and eventually calculating the maturity indices using Equations (1) and (2).

(1) $$FMI=\frac{\left(Fresh\;seed\;weight\right)}{\left(Fresh\;hull\;weight\right)}$$

(2) $$DMI=\frac{\left(Dried\;seed\;weight\right)}{\left(Dried\;hull\;weight\right)}$$

FMI and DMI values were generally reported to increase with maturity, but ranges varied for different cultivars. For example, Florigiant had a maximum DMI value of 3.19 and 3.02 in two years of testing in 1974 and 1975, while Florunners’ DMI ranged from 4.09 to 4.39 [62]. These values were also reported to have negatively correlated with AMI and used as a good indicator to determine yield and quality of Virginia-type peanuts [62,63]. The indeterminate growth habit of peanuts poses challenges for the effective use of this method, as the variability in canopy greenness may not reliably indicate maturity status and can influence decisions on sampling location and time, amounting to subjectivity and possible inaccuracies in evaluations. To mitigate this, the defined temporal schedules of standardized grid-based, random, or stratified sampling across field variability zones, combined with the integration of GDD accumulation thresholds, may improve representativeness and reduce reliance on canopy appearance [64]. Additionally, the process of sampling at different dates, followed by separating pods and obtaining fresh and dry weights, is time-consuming and typically requires at least a week to complete the evaluation of multiple samples collected during a single timestamp.

### 5.5. Williams and Drexler or Hull Scrape Method

**Figure 3:**
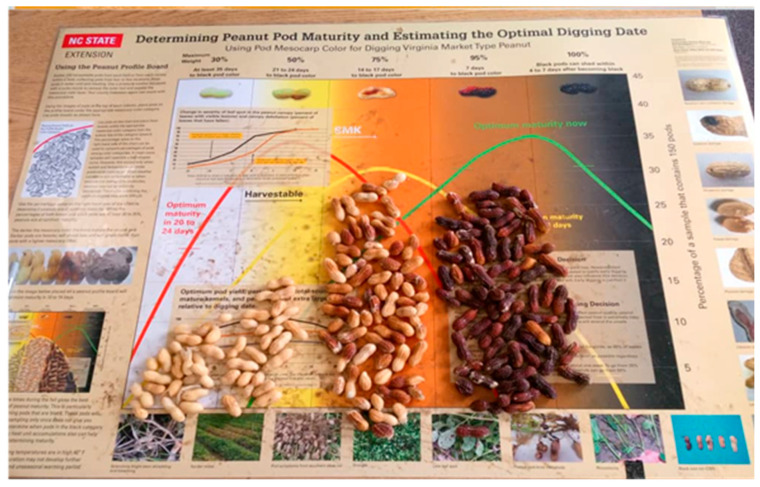
The example of the peanut maturity profile board used for the manual-visual grading of peanut pods for maturity based on the color of Virginia-type peanut cultivars that transition from white to yellow, orange, brown, and black. These gradings are used to calculate the peanut maturity index. The red curve on the chart indicates that peanuts are about 20–24 from optimum maturity, yellow curve indicates 10–14 days from optimum maturity while green curve indicates that optimum maturity is current.

The most widely used direct method for assessing peanut maturity is the Williams and Drexler’s hull scrape method [10], which relies on determining maturity based on the mesocarp color that changes from white to black as pods progress toward maturity. Three–four peanut plants are randomly dug using shovels from one or two locations in the field, and pods are detached manually to obtain about 100–150 pods. Next, pods are subjected to pressurized-water, sand or glass beads-based blasting where the outer layer (exocarp) of the pods is stripped off and the mesocarp layer is exposed. Blasted pods are then sorted by color (white, yellow, orange, brown, and black) by placing them on the maturity profile board (MPB, Figure 3). Each color class represents specific developmental stages, where white indicates newly developed pods while the black color indicates 100% matured pods.

Once all the pods are placed on the MPB, the visual approximation of number of days to maturity is made. The MPB, developed by Williams and Drexler, was used for manually classifying runner, Spanish, and Valencia types of peanut pods into six color classes, namely, white, yellow-1, yellow-2, orange, brown, and black [17]. Later, North Carolina State University introduced an updated MPB with five color classes, i.e., white, yellow, orange, brown, and black, for classifying Virginia-type peanut pods (Figure 3). Manual classification helps in estimating the number of days to optimum maturity but does not help quantify the exact maturity status. To meet this requirement, Rowland et al. [65] reported the peanut maturity index (PMI) as a peanut maturity quantifier calculated based on the proportion of matured pods (brown and black) to the total number of pods in a sample (Equation (3)) [65].

(3) $$PMI=\frac{\left(Number\;of\;brown\;pods+Number\;of\;black\;pods\right)}{\left(Total\;number\;of\;pods\;in\;the\;sample\right)}$$

As a standard practice, peanuts with a PMI of ≥70% are said to be ready for an optimal harvest that balances yield and pod retention. The PMI strongly correlates with key economic factors such as yield, grade, and net market value, making it a reliable and practical tool for determining the best harvest time [64]. The MPB is particularly useful for fields with bimodal maturity distributions (distinct immature/mature clusters) and requires 25–30 min per sample starting with pod sampling from field to estimate harvest windows [64]. While the MPB color classification enables equipment-free maturity assessment, challenges include labor-intensive pod preparation, subjective color interpretation, and undefined sampling protocols for field variability. The process is time-consuming and may require multiple sample collections and campaigns for accuracy and field-level representation [64,65].

### 5.6. Thermal Time Method

In 1989, Ketring and Wheless [66] developed the thermal time method, which quantifies the impact of temperature on peanut plant development and provides a framework for estimating peanut pod maturity. This method determines the significant impact of environmental factors on the peanut plant development rate, including temperature, soil composition, and irrigation. It employs the classification of plant development phases as described by Boote [9], facilitating a detailed understanding of both vegetative and reproductive growth stages via thermal time. The thermal time expressed in day-degrees (°Cd, Equation (4)) serves as a more precise parameter than calendar days for tracking the peanut phenological development.

(4) $$Thermal\;time\left(t_{n}\right)\left({}^{\circ}Cd\right)=\sum_{i=1}^{n}\left[\frac{{Ti}_{max}+{Ti}_{min}}{2}-T_{b}\right]$$

where *Ti_max_* and *Ti_min_* are the maximum and minimum temperatures of the day, *T_b_* is the peanut base temperature, *i* indicates a day, *n* is the number of days in the growing period, and *t_n_* is the sum of the total *n* day-degrees (thermal time) [67]. A base temperature (*T_b_*) of 13 °C was used to estimate thermal time, which strongly correlates with the vegetative and reproductive development stages of plants [68]. Ketring and Wheless [66] studied the relationship between thermal time and peanut pod maturity over three years using two types of peanuts, “pronto” (Spanish) and “OK-FH15” (Virginia). They discovered a strong relationship between accumulated day-degrees (°Cd) and maturity stages. For example, pronto started flowering at 313 °Cd and OK-FH15 at 360 °Cd, with 50% flowering occurring at 410 °Cd for pronto and 498 °Cd for OK-FH15. The total heat accumulated during the season ranged from 1456 °Cd to 1672 °Cd, showing consistent growth even in different environments. These research findings show that thermal time is one of the effective ways to measure crop growth [34,67]. Instead of using daily GDD, the study used accumulated GDD (AGDD) for a more complete understanding of crop growth. The AGDD adds up daily GDD over the growing period, capturing the total heat experienced by the crop. This makes AGDD helpful for estimating maturity of the pods and identifying the best digging time in different growing conditions [66].

The thermal time or AGDD method uses accumulated heat units not just to estimate plant development but also for predicting crop growth stages with added benefits in irrigation scheduling, harvest optimization with identifying precise digging dates, genotype performance comparison, and estimating net returns. The method however has limitations, including sensitivity to water deficit conditions, which can alter plant development rates and reduce estimation accuracies. Environmental factors like photoperiod and soil conditions may also be needed to broaden the applicability of this method across diverse climates and peanut genotypes. Different peanut varieties may also have varying thermal time requirements, necessitating calibration for each cultivar. Furthermore, this method may not be able to account for in-field spatial variations in crop development as the monitoring of microclimate variations within the field could be highly difficult and expensive that would need the installation of multiple weather sensors in a single field. Despite these constraints, the thermal time method has the potential to be a powerful tool for peanut crop management when used in conjunction with other high-resolution environmental data and crop monitoring techniques.

Conventional peanut maturity estimation methods, while accurate, are low-throughput and limited in field-scale applicability. This necessitates advanced, high-throughput approaches that support timely and robust harvest decision-making [69]. Recent advances in proximal and remote sensing, along with optical and spectral imaging and modern data processing such as AI, offer scalable solutions for field-level maturity assessment; however, research in this area remains limited and is explored in the following sections.

## 6. Advanced Methods and Technologies for Assessing Peanut Maturity

This section discusses recent technological advancements in proximal and remote sensing technologies along with optical sensing and spectral imaging sensors as well as advanced data processing methods that offer capability for efficient, scalable, and large-scale peanut maturity assessments over conventional approaches.

### 6.1. Proximal Sensing Systems

Proximal sensing is an advanced non-invasive technique that makes assessment of a target object from a very close non-contact observation using visible range (RGB), multispectral, hyperspectral, thermal, and spectroscopy-type sensors, among others. Key proximal sensing techniques employed for peanut maturity assessments include spectral reflectance, near-infrared (NIR) spectroscopy, and visible, multispectral, and hyperspectral imaging technologies. Such techniques have been used to evaluate pod characteristics such as chemical composition, surface color, and internal and external structure properties, among others. Such assessments, mostly capable of being real-time and of high-resolution, offer opportunities for the precise estimation of peanut maturity. So far, only a few of such studies have been found that used proximal sensing or imaging to assess pod maturity.

The proximal sensing approaches described in this section can be broadly categorized based on (i) the type of spectral information used and (ii) the target of measurement. Some studies rely on raw reflectance features or specific wavelength responses (e.g., spectral peaks and absorption features), while others employ derived spectral indices or multivariate modeling approaches (e.g., PLSR) applied to spectral data. In addition, a distinction exists between methods applied directly to excavated pods (often following blasting or preparation) and those based on aboveground canopy reflectance measurements for indirect maturity estimation.

Studies have used a hand-held multispectral radiometer to characterize peanut maturity using subtle variations in canopy reflectance features. Reflectance in the NIR region (830–850 nm) was reported to showcase a strong correlation with pod maturity for being able to capture physiological and structural changes in the plant canopy [70]. However, more studies in this direction were warranted. In the same year, spectroradiometer (350–2400 nm) was used to quantify peanut maturity using canopy reflectance for Virginia-type peanuts, and strong correlations (R^2^ up to 0.66) were observed [71]. Similarly, hand-held spectrometers have been used to detect maturity-related spectral responses in the mid-NIR region (1365–2335 nm), where wavelengths associated with canopy and leaf biochemical properties provide complementary information for maturity characterization, indirectly linked to pod maturity [72]. These canopy-based approaches utilize raw reflectance features derived from aboveground plant observations for indirect maturity estimation.

In contrast to canopy-based approaches, the study by Windham et al. [73] used visible–NIR (Vis-NIR, 400–1100 nm) spectroscopy to analyze reflectance features from the basal and dorsal regions of the pods. Partial least squares regression (PLSR) models were then formulated to predict maturity classes, which were then used to estimate the optimal days to dig peanuts. Results showed strong correlations between reflectance data and maturity stages (R^2^~0.94) with ±3 days of alignment with traditional MPB estimates. Key spectral wavelengths in the 640 nm and 976 nm regions were identified as the best ones to be determinants of the pod maturity. Visible-range proximal (RGB) imaging with color-based classification techniques has also been evaluated on mesocarp-exposed peanut pods for maturity assessments for a range of runner and Virginia-type cultivars in Florida [74], South Carolina [75], and Virginia [76]. The classification accuracies were reported in the ranges of 92–94%, R^2^ values in the ranges of 0.19 to 0.98, and root mean square error (RMSE) in the ranges of 2.5–4%. The study by Colvin et al. [74] also reported that the pods had to be in certain orientations to capture their saddle region for higher and stable estimation accuracy compared to randomly captured regions on the pod that led to variable accuracies in classification. Vis-NIR hyperspectral imaging (HSI) in the wavelength ranges of 400–1000 nm was shown to be promising for classifying peanut maturity without the removal of the exocarp layer from pods [77]. Reflectance features were derived, and the NIR range was found highly effective in distinguishing mature and immature pods with accuracies up to 93% across different peanut cultivars and growing seasons. These approaches are primarily based on the imaging of excavated pods (including RGB and hyperspectral imaging after hull scraping or pod blasting), rather than intact-plant observations.

Proximal sensing methods are objective and have demonstrated high accuracy; however, they often require manual sampling and pod blasting as well as pods to be at certain orientations before evaluations; therefore, such methods may not be called entirely non-invasive or high throughput. However, canopy reflectance-based methods showcase possibilities for non-invasive field-scale assessments. Furthermore, most of the above-mentioned methods require manual pod sampling from the field followed by blasting, which tends to add labor-intensiveness to the methods. Even the number of collected samples may not be sufficient, and their selection could be subjective such that spatial accuracy in field-variability representation appears to be a bigger challenge. Critically, most of these methods offer restricted adaptability by the peanut growers due to sensor and setup costs. With the expansion of high-resolution remote sensing, the scale of crop monitoring has increased substantially. While proximal sensing remains essential for controlled maturity assessment, calibration, and validation, its scalability for field-level peanut maturity assessment is limited. Nevertheless, canopy-based proximal sensing, when supported by standardized sampling protocols, continues to play a critical role in calibration and validation to enhance the field representativeness of large-scale approaches.

### 6.2. Remote Sensing Systems

Remote sensing systems typically driven by occupied and unoccupied aerial systems (UASs) and satellite platforms have emerged as powerful transformative technology for agricultural applications over the last five decades [78]. It was the launching of the first satellite, Landsat-1, in 1972 that marked the revolution for agricultural monitoring and precision agriculture. It was also the first satellite to provide multispectral images for large-scale crop monitoring [79]. This technological breakthrough enabled investigation into agricultural traits such as vegetation health, soil conditions, and crop growth over large areas with better accuracy. Later, MODIS and Sentinel satellites further enhanced the scope for agricultural monitoring through enhanced capabilities and improved data quality at higher spatiotemporal and spectral (10 m to 1 km/pixel) resolutions [80,81]. Over the past couple of decades, high-spatial resolution data for agricultural applications has been delivered by the UASs, and low-orbital satellites provide plant-to-field-level details with high granularity (mm to 3 m/pixel). Such platforms deploy a suite of sensors across various regions of the electromagnetic spectrum to detect reflected or emitted energy, far from the ground [82]. Typical to agriculture, remote sensing has proven its significance for monitoring crop health and growth status at high-throughput capacity with a wider coverage and capability to provide the estimation of spatial variabilities in the field [83,84]. These capabilities provide a valuable opportunity for the precision mapping of peanut maturity built upon the exploration of proximal sensing-based reflectance features that were noted to be significant.

Remote sensing-based approaches using satellite and UAS platforms have further expanded these capabilities for non-invasive peanut maturity assessment at larger spatial scales. Several studies have explored the use of high-resolution multispectral imagery (2–5 m/pixel) to derive VIs and relate them to ground-based maturity indicators such as the peanut maturity index (PMI) obtained using hull scrape and MPB methods [17,72,85,86,87]. The PMI, derived from these destructive methods, is used as a ground-truth reference for regression-based model training. Remote sensing data captures aboveground canopy characteristics, and statistical/ML models are then employed to learn the relationships between these canopy features and pod maturity, enabling the indirect estimation of PMI at the field scale. Across these studies, VIs derived from the visible and NIR spectral regions, including indices such as NDVI, GNDVI, SAVI, NLI, MSAVI, MCARI, and SR, as well as red-edge-based metrics such as NDRE, SRRE, MNLIRE and NLIRE, demonstrate moderate-to-strong relationships with peanut maturity (Table 3, [85,88,89,90,91,92,93,94]).

Beyond the multispectral modalities spanning Vis (400–700 nm), NIR (700–1300 nm), and red-edge (680–750 nm) spectral regions, most directly represented in the current peanut maturity studies, a broader sensing space, in which radar-based systems such as SAR, operating in the microwave region and including bands such as X-band (~8–12 GHz; wavelength ~2.5–3.75 cm), C-band (~4–8 GHz; ~3.75–7.5 cm), L-band (~1–2 GHz; ~15–30 cm), and P-band (~0.3–1 GHz; ~30–100 cm), can contribute to complementary structural and all-weather observation capability and hyperspectral imaging, spanning the visible to shortwave infrared region (VSWIR; 400–2500 nm), and can provide narrow-band biochemical information, and its fusion with depth-sensing technologies (750–1550 nm) such as LiDAR, RGB-D, or photogrammetric 3D reconstruction may further strengthen structural and canopy-level interpretation of variability related to peanut maturity patterns at the field scale in future crop monitoring applications. These remote sensing features have enabled the use of statistical and machine learning models such as Gompertz functions, partial least squares regression (PLSR), partial least squares discriminant analysis (PLSDA), and artificial neural networks (ANNs) for maturity estimation. For instance, satellite-based PlanetScope imagery was used to estimate peanut maturity in Brazil and generate spatial maturity maps using vegetation indices derived from multispectral data [17]. High-resolution satellite-based multispectral imagery and associated VIs from QuickBird platform have also been used to assess peanut maturity and aflatoxin risk in peanut crops of different varieties in Australia [72]. Similarly, UAS- and occupied aircraft-based multispectral imagery (0.08–5 m/pixel) has been used to monitor peanut maturity progression and derive multiple VIs that show strong correlations with maturity indicators, with red-edge-based indices often demonstrating improved sensitivity to canopy physiological changes during late growth stages [84,85,87].

Comparative investigations further indicate that satellite-, occupied aircraft- and UAS-based platforms can support peanut maturity estimation, although each offers different advantages. Satellite platforms provide broader spatial coverage suitable for large-scale and repeated monitoring but are constrained by cloud interference and coarser spatial details, whereas UASs and occupied aircraft platforms offer higher spatial resolution and greater flexibility in acquisition timing for precision mapping within fields. The reviewed peanut maturity studies are dominated by multispectral and red-edge observations, while hyperspectral, thermal, radar, and structural sensing modalities remain more prospective than established in this application. Calibration to destructive ground-truth maturity measurements remain critical across platforms, and transferability across cultivars, environments, and seasons is still limited in the current literature. Studies integrating multispectral imagery from the PlanetScope CubeSat platform with machine learning models such as artificial neural network (ANN) has shown improved maturity estimation compared to traditional regression (PLSR and PLSDA) approaches, particularly when integrating environmental variables and management conditions [16]. These findings highlight the potential of combining high-resolution remote sensing with data-driven models for operational maturity monitoring. However, several investigations have also reported reduced predictive accuracy during early growth stages, suggesting that remote sensing-based maturity estimates are more reliable closer to harvest when canopy spectral signals better reflect pod development.

Most of the synthesized studies have primarily focused on regular runner or market-type cultivars, often relying on a limited dataset size and a couple of agroclimatic conditions used for growing the same type of peanuts, and as obtaining ground-truth data is highly challenging. Findings of such studies are encouraging and need further expansion and robust testing for numerous other cultivar types, larger datasets for generalization, and evaluations under multiple agroclimatic conditions across the peanut-growing regions. Including weather information in estimating peanut maturity may further enhance the accuracy and robustness of maturity estimation as well as forecasting based on weather forecasts, given when peanut maturity is critically affected by weather factors. It is also worth noting that acquiring high-resolution imagery from UAS platforms or low-orbital satellites demands costs, with the latter having a lower data availability. In such situations, open-access and moderate-resolution satellite imagery could be helpful as that can enhance the end-user adaptability of peanut maturity estimation tools based on such datasets. It must also be noted that satellite imagery can be highly affected by cloud covers while flexible data acquisition opportunities with UAS platforms can eliminate such vulnerabilities [95]. Therefore, a trade-off between data quality, spatial resolution, and costs would be required when envisioning farmer-adoptability of peanut maturity estimation techniques for high precision. In practical applications, a satellite-based maturity assessment is additionally constrained by cloud interference, revisit limitations, and dependence on high-quality ground-truth calibration. These limitations may be partly reduced through flexible UAS-based acquisitions, multi-date observations, and integration with ground-based measurements for calibration and validation.

### 6.3. Artificial Intelligence (AI) for Peanut Maturity Estimation

Over the past decade, agriculture and related research have seen significant transformation from manual methods to evolving automated methods supported by technologies such as AI. AI is no longer futuristic but is already adopted for crop management, yield predictions, crop assessments, and improved decision-making, as well as driving automated operations on ground such as spraying, irrigation, and soil preparation, among others. AI is forming a new modern-day farming framework that feeds on data flowing from sensors (invasive, proximal or remote) and provides actionable insights or actual actions on the ground. Characterizing crop growth and development depends on numerous agroclimatic factors, simultaneously handling of which is highly complicated [95]. AI with ML and DL algorithms emerges to address this challenge by having capabilities to analyze large datasets, identify complex patterns, and make accurate estimations and predictions [96,97,98,99].

#### 6.3.1. Machine Learning Applications

**Figure 4:**
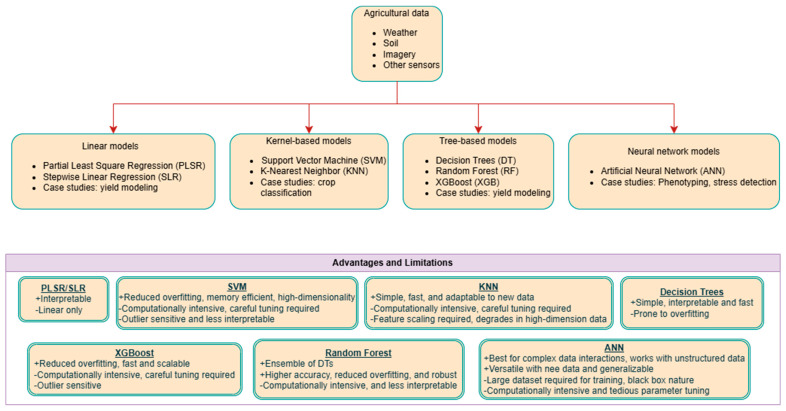
Conceptual overview of machine learning algorithms applied in peanut yield and maturity estimation, highlighting general strengths and limitations in field-scale agricultural modeling.

Supervised ML approaches have increasingly been applied to predict peanut yield, biomass, and maturity using integrated agronomic, climatic, and remote sensing datasets. Recent studies have demonstrated the feasibility of ML approaches for both regression-based yield estimation and quantitative maturity prediction at the field scale. These approaches can be broadly grouped into linear, kernel-based, tree-based, and neural network approaches (Figure 4). Across studies, tree-based, kernel-based, and neural network models consistently outperform simple linear regressions when heterogeneous agronomic and spectral inputs are integrated.

Multi-source yield modeling frameworks integrating field management, soil, weather, and remote sensing variables have demonstrated improved predictive robustness compared to models relying solely on vegetation indices. For example, Scarpin et al. [100] evaluated 18 ML algorithms across multiple variable groups using data from over 200 farms in Georgia, USA, and reported that tree-based models and support vector approaches achieved the lowest prediction errors (RMSE = 816 kg ha^−1^ for yield). Importantly, leave-one-site-year-out validation demonstrated spatial generalization capacity under heterogeneous production environments. Phenology-aware modeling has also proven critical for improving yield prediction accuracy. Hou et al. [101] showed that incorporating multi-temporal vegetation indices representing both early biomass accumulation and late-stage assimilate transfer significantly improved predictive performance (R^2^ up to 0.82) compared to models relying solely on the maximum NDVI. Similarly, UAS-based multispectral imagery combined with ensemble ML approaches achieved strong predictive performance, particularly when maturity-stage spectral features were included [95], although evaluations were limited to a single growing season.

Limited studies have evaluated peanut maturity using ML approaches. Early investigations demonstrate the feasibility of regression-based modeling using spectral reflectance data derived from both excavated pods (pod-based approaches) and aboveground canopy observations (canopy-based approaches). In practical terms, algorithm suitability is closely linked to input type: PLSR has mainly been applied to spectral reflectance data, while ANN- and other ML-based approaches have more often been used with VIs and fused agronomic or environmental inputs for field-scale maturity estimation. Using a pod-based approach, Windham et al. [73] demonstrated that the PLSR algorithm with Vis-NIR reflectance spectroscopy data achieved an accuracy with R^2^ of 0.94 between actual and estimated maturity. PLSR was also used in a canopy-based approach by Abd-El Monsef et al. [86] with UAS-based multispectral imagery-derived VIs to predict peanut maturity that were able to perform well after 60 days of plantation. ANN models with multi-layer perceptron and radial bias function were used in canopy-based approaches by Santos et al. [87] and Souza et al. [16] on UAS-based as well as high-resolution satellite-based multispectral imagery to estimate peanut maturity under irrigated and rainfed conditions and achieved high accuracy (R^2^ of 0.91, RMSE of 0.062, and MAE of 0.05), outlining NDRE and NDVI features to be most effective. More recent developments extend beyond single-output maturity prediction. Oliveira et al. [102] introduced a canopy-based multi-target regression framework capable of simultaneously predicting the peanut biomass index and maturity index (PMI) using satellite-derived spectral reflectance, reporting maturity prediction errors below 10%. This represents a transition from proof-of-concept maturity classification toward quantitative field-scale maturity mapping. In addition to regression-based frameworks, hyperspectral feature reduction combined with traditional ML classifiers has also been explored for maturity classification. Tushar et al. [103] demonstrated a pod-based approach in which principal component analysis (PCA)-derived spectral features coupled with an RF model achieved comparable performance to computationally intensive linear unmixing approaches in low-cost maturity detection systems. Despite promising accuracy levels, these maturity-focus ML explorations were, however, evaluated by single cultivars and a maximum of two agroclimatic growing conditions. In addition, weather factors that are critical for determining peanut growth and maturity were not considered. Addressing these factors may very well enhance robustness and applicability of ML-based approaches for peanut maturity estimation at a global level. From a practical standpoint, ML-based maturity estimation may also be affected by overfitting when models are trained on limited samples, narrow cultivar sets, or restricted agroclimatic conditions, which can reduce transferability across sites and seasons. Broader multi-environment datasets, independent validation sets, and testing across diverse cultivars and production conditions are needed to improve model robustness for operational use. Overall, canopy-based ML approaches dominate field-scale maturity estimation due to their ability to integrate vegetation indices and agronomic and climatic variables within structured modeling frameworks, while pod-based ML approaches provide high-accuracy measurements at the sample scale but remain labor-intensive and limited in scalability.

#### 6.3.2. Deep Learning Applications

The DL aspect of AI is the next-frontier technology that enables automated analysis from digital images and videos and then comprehends features for identification and classification applications. DL approaches have increasingly been explored for peanut maturity estimation due to their ability to model complex non-linear relationships and automatically learn discriminative representations from high-dimensional spectral and imaging datasets. Unlike classical regression or tree-based ML approaches that rely heavily on handcrafted vegetation indices, DL architecture enables hierarchical representation learning (HRL) from raw spectral or image inputs, potentially reducing subjectivity and improving generalization across heterogeneous production systems.

Among field-scale maturity applications, ANN models integrating remote sensing and climatic information have demonstrated strong predictive capability. Santos et al. [87] showed that ANN models incorporating canopy-based VIs and adjusted GDD (aGDD) substantially outperformed linear and multiple regression approaches, achieving R^2^ values of 0.91 under irrigated conditions. Similarly, Souza et al. [16] integrated UAS and satellite imagery within multilayer perceptron (MLP) and radial basis function (RBF) architectures to estimate the peanut maturity index (PMI) at the canopy level, reporting R^2^ values exceeding 0.87 with a low prediction error. These findings underscore the ability of neural networks to capture non-linear interactions between canopy reflectance and thermal accumulation. Recent advancements in DL architecture have extended ANN-based maturity modeling to cross-field generalization scenarios. Oliveira et al. [104], in a canopy-based DL framework, combined PlanetScope orbital imagery, accumulated degree-days (ADD), and neural networks (MLP and RBF) to estimate peanut pod maturity across two commercial fields and growing seasons. While within-field validation achieved R^2^ values between 0.90 and 0.93, cross-site generalization without retraining yielded moderate performance (R^2^ = 0.59–0.64), highlighting both the strength and limitations of current DL maturity models under heterogeneous agroclimatic conditions, demonstrating that coupling climatic and spectral variables within ANN architectures improves interpretability and practical relevance for harvest decision support [102].

At the pod and seed scale, traditional image processing combined with DL architecture has been explored for automated maturity classification. Bindlish et al. [76] used pod-based DL approach with RGB images and converted them to HSV and LAB color spaces to enhance feature extraction. Thresholding through morphological operations was then used to segment the mesocarp regions of the pods. Next, the mesocarp color of pods was categorized into 10 groups representing different maturity stages. The method achieved a recall and a precision of 66.5% for classifying pods into seven maturity stages and achieved an accuracy of 92.5% for identifying mature pods in the brown-black category. Similarly, pod-based color classification using unsupervised approaches was employed by Colvin et al. [74] and Liang et al. [75] to classify peanut pods into five color classes. Such methods, however, require substantial image preprocessing and correction of illumination effects to minimize noise on top of the extensive sampling of pods in the fields or manual pod preparation before imaging and analysis, making them tedious and less practical for peanut growers. Recently, Balasubramaniyan and Navaneethan [105] proposed a seed-based Hyper Spectral Invariant Scaled Feature Selection (HSISFS) framework coupled with an Adaptive Dense Net Recurrent Neural Network (ADNRNN) to classify mature and non-mature peanut seeds. By leveraging feature scaling and recurrent learning structures, the approach achieved a classification accuracy of approximately 89%, demonstrating the feasibility of DL-based hyperspectral feature learning for objective maturity assessments. Such hyperspectral DL frameworks reduce reliance on manual exocarp removal and subjective color interpretation, offering potential for automated grading systems.

Despite promising performance, DL applications for peanut maturity estimation remain constrained by limited cultivar diversity, restricted geographic validation, and relatively small training datasets. Most studies have been conducted using one or two cultivars under specific production environments, and the temporal sequence modeling of maturity progression using advanced recurrent or transformer-based architectures has not yet been extensively explored. Larger and more diverse training datasets, cross-site or cross-season validation, and transfer learning strategies may help improve the practical applicability of DL-based peanut maturity estimation. Furthermore, large-scale operational deployment and transferability across regions and seasons remain challenging. Future research could explore sequential learning architectures such as Long Short-Term Memory (LSTM) or Gated Recurrent Unit (GRU) networks, transformer-based temporal models such as Vision Transformers (ViTs) or Swin Transformers, spatiotemporal models such as 3D-Convolutional Neural Networks, CNN–RNN hybrid frameworks (e.g., CNN-LSTM or CNN-GRU), physics-informed neural networks integrating crop growth variables, transfer learning approaches using pretrained models such as AlexNet, ResNet, DenseNet, MobileNet, VGGNet, EfficientNet, Inception, or You Look Only Once (YOLO), self-supervised learning methods such as SimCLR or masked autoencoders, and multimodal data fusion architectures with self-attention mechanism (e.g., attention-based multi-layer fusion networks) combining spectral, climatic, and environmental variables to improve the robustness and scalability of peanut maturity monitoring systems.

Table 4 presents a structured comparison of various algorithms used for peanut maturity estimation, clearly distinguishing algorithm classes, data types, performance, complexity, advantages, scenarios, and limitations. Pod-based approaches, particularly PLSR with Vis–NIR data and hyperspectral DL models, achieve the highest accuracy at the sample scale but are labor-intensive and not scalable. For field applications, tree-based ML, ANN, and ensemble models provide the best balance of accuracy, robustness, and scalability by integrating multispectral imagery, vegetation indices, and climatic variables. Deep learning combined with multi-source data fusion and phenology-aware modeling offers strong future potential by capturing non-linear relationships and crop growth dynamics. However, adoption remains constrained by limited multi-environment datasets, narrow cultivar diversity, and poor cross-site transferability, along with practical challenges related to sampling, calibration, cost, and operations. These limitations highlight the need for an integrated Agriculture 5.0 framework that connects sensing, analytics, and grower-oriented decision support, motivating the development of high-throughput maturity mapping systems. Along this direction a prospective framework informed by the reviewed literature is outlined in the next section for translating current sensing and AI capabilities into operational peanut maturity assessment systems.

## 7. Prospective Framework for High-Throughput Mapping of Peanut Maturity in Agriculture 5.0

The following section is presented as a prospective framework informed by the reviewed literature and is intended to outline future directions for translating current sensing and AI capabilities into operational peanut maturity assessment systems.

### 7.1. Limitations of Traditional Peanut Maturity Assessment Methods

Over the past several decades, numerous approaches have been explored to estimate peanut maturity, ranging from traditional invasive methods to emerging non-invasive techniques. However, many of these approaches remain subjective, labor-intensive, and time-consuming, often requiring large sample sizes to generate field-representative estimates. Consequently, their applicability in commercial production systems is limited. Conventional methods such as hull scrape or pod blasting followed by grading using the maturity profile board provide reliable maturity assessments but are destructive, require skilled labor, and are difficult to scale across large production fields. In contrast, research based on spectral reflectance and vegetation indices has demonstrated substantial potential for non-invasive and high-throughput maturity assessments at larger spatial scales. The increasing availability of remote sensing platforms such as UASs has further enhanced the ability to capture high-resolution spatial information on crop growth and canopy conditions [106].

### 7.2. Emerging Role of AI and Remote Sensing

Although limited, recent studies have explored the integration of UAS- and satellite-based multispectral imagery with ML algorithms to estimate peanut maturity. These approaches highlight the growing role of AI and remote sensing technologies in monitoring crop development and supporting precision agriculture where advances in DL and AI-driven analytics have significantly expanded the capability to process complex agricultural datasets and extract actionable insights for crop management [107,108]. Despite these advancements, many existing studies remain limited in their scope for operational deployment. Although these studies demonstrate the potential of sensing- and AI-based approaches for peanut maturity estimation, the broader integration into operational decision-support systems remains largely prospective. From a sensing standpoint, this limitation also reflects an insufficient comparison across acquisition timing, spectral configuration, platform choice, calibration strategy, and field validation design in the current peanut maturity literature. In particular, several approaches do not adequately incorporate (1) weather variables influencing crop growth and physiological development, (2) variability across field environments and management systems, (3) cultivar-specific growth characteristics, and (4) mechanisms for translating maturity predictions into actionable decision-support tools for peanut growers. Feedback from producers further indicates a clear demand for systems capable of guiding harvest initiation, optimizing resource allocation, and supporting crop insurance and financial planning decisions.

### 7.3. GeoAI for Peanut Maturity Mapping

Within the broader paradigm of Agriculture 5.0, digital agriculture systems are increasingly characterized by the integration of AI, autonomous sensing technologies, and interconnected decision-support platforms designed to support human-centered agricultural management. These systems rely heavily on data-driven approaches and large-scale analytics to transform agricultural decision-making processes [109,110]. A critical technological enabler in this context is Geospatial Artificial Intelligence (GeoAI), which integrates spatial data science with ML to extract geospatial knowledge from complex datasets [111]. GeoAI enables the fusion of multi-source datasets including remote sensing observations, environmental measurements, and spatial analytics to generate predictive insights for crop monitoring.

Modern sensing platforms including UASs, satellites, and occupied aircraft systems equipped with multispectral or hyperspectral sensors can capture detailed spatial information related to crop canopy structure, vigor, and physiological status. When combined with environmental variables such as temperature, precipitation, GDDs, and soil moisture, these systems generate multi-dimensional datasets capable of capturing both crop status and environmental drivers of development. GeoAI models can then analyze these datasets to estimate peanut maturity progression and predict the number of days remaining until optimal harvest.

### 7.4. Integrated Digital Pipeline for Peanut Maturity Monitoring

**Figure 5:**
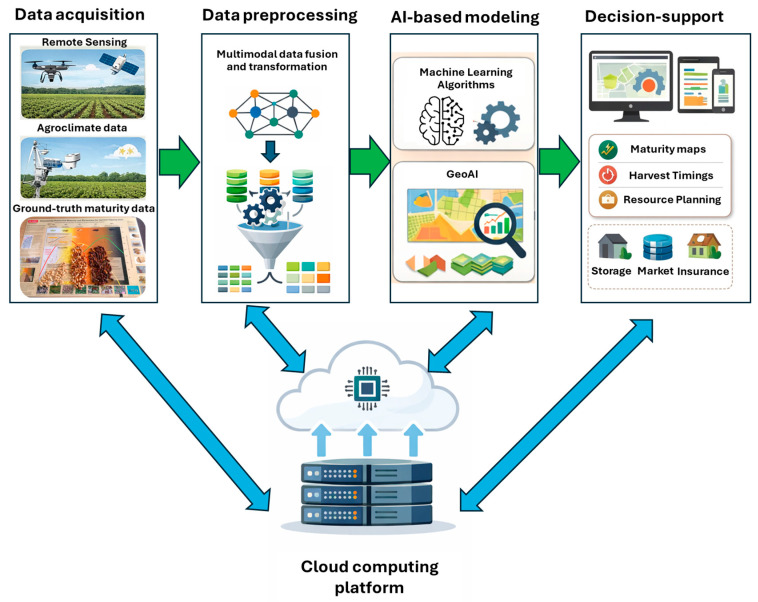
Schematic workflow of an Agriculture 5.0-enabled decision-support system for precision peanut harvest management, illustrating the integration of ground-truth maturity sampling with multi-source sensing, cloud-based analytics, and intelligent advisory tools to optimize maturity assessment, harvest timing, and farm-level decision-making.

To operationalize such capabilities, an integrated data and analytics pipeline is required (Figure 5). Accordingly, the pipeline presented below should be interpreted as a conceptual synthesis and forward-looking implementation pathway rather than as a fully established workflow already validated in the literature. The pipeline begins with multi-source data acquisition, where spectral imagery is collected through UAS- or satellite-based multispectral platforms via application programming interfaces (APIs) or automated ingestion systems. In parallel, agroclimatic variables including temperature, humidity, precipitation, soil moisture, soil temperature, and solar radiation are integrated to capture environmental drivers of crop growth. The practical value of this pipeline depends on the aligning sensor choice, acquisition timing, and calibration strategy with the maturity signal being targeted, from canopy reflectance, vegetation indices, or integrated environmental response.

Previous studies by Rowland et al. [65] and Santos et al. [87] highlighted the importance of incorporating environmental factors such as photoperiod and GDDs in peanut maturity estimation models. Integrating these variables can significantly enhance model robustness and predictive accuracy across diverse production environments. Ground-truth data collection represents another critical component of the pipeline. To improve practical feasibility, ground-truth maturity sampling should be designed using stratified and reduced-sample approaches that capture field variability while minimizing labor and cost. Such sampling may combine limited destructive pod sampling at representative field zones with canopy-based observations to support model calibration more efficiently. Although challenging, reliable maturity datasets can be obtained using destructive methods such as hull scrape or pod blasting followed by grading with the maturity profile board. To ensure model generalizability, these datasets should be collected across multiple environments, field locations, peanut cultivars, and growth stages throughout the season. Recording the number of days to optimum maturity for each graded sample further enables the temporal modeling of maturity progression. Once these datasets are assembled, AI and ML models can be trained to estimate maturity status and predict the remaining days until optimal harvest. For broader operational use, model transfer across regions and production environments may be improved through transfer learning, recalibration with limited local ground samples, and multi-view, multi-model or multi-source data fusion strategies that combine spectral, thermal, climatic, and management information.

### 7.5. Decision-Support Systems for Grower-Oriented Applications

To deliver actionable insights to end users, trained models must be integrated within geospatial data environments where predictions can be applied to remotely sensed imagery and environmental datasets. The resulting outputs can then be disseminated to growers through user-friendly graphical interfaces capable of generating geospatial maturity maps and estimates of days to optimal harvest. Such systems could allow users to access relevant data inputs and retrieve maturity-related information through simple interactions. Additional decision-support features may further enhance the utility of these tools by incorporating logistical considerations such as storage availability, nearby marketing locations, commodity prices, supply-chain logistics, and crop insurance information.

### 7.6. Cloud-Based Infrastructure for Scalable Deployment

Operational deployment of robust peanut maturity mapping systems requires infrastructure capable of managing large volumes of remote sensing, environmental, and field datasets. This is particularly important for sensing-driven maturity workflows that must integrate multi-platform imagery, repeated acquisitions, calibration samples, and model outputs within a single operational environment. Cloud-based computing platforms such as Google Earth Engine, Amazon Web Services or Microsoft Azure can support large-scale geospatial processing, while spatial database systems such as PostgreSQL with PostGIS extensions enable efficient storage, querying, and analysis of spatial and non-spatial data. For practical deployment, cloud platforms should be designed with modular and cost-aware workflows, including selective data storage, automated processing pipelines, and use of open-access imagery where possible to reduce operational expenses. A combination of cloud computing for large-scale processing and lighter user-facing tools for visualization may offer a more affordable deployment pathway. Similar data-intensive decision-support infrastructure is widely used in sectors such as logistics, energy systems, and environmental monitoring, where cloud computing and scalable analytics pipelines are routinely used to process large volumes of sensor data for operational decision-making. These examples demonstrate the feasibility of integrating distributed sensing, cloud computing, geospatial analytics, and user-oriented dashboards for agricultural applications such as precision peanut maturity mapping and harvesting.

### 7.7. Future Opportunities for Digital Harvest Management

If supported by adequate resources, such systems could potentially be deployed as open-source platforms or offered through low-cost subscription models. However, extensive multi-environment validation will be required before operational deployment to ensure reliability across production environments and protection against emerging cybersecurity threats. Beyond peanut maturity monitoring, the proposed framework could also be adapted for broader crop management applications, including planting, crop protection, and harvest scheduling across multiple cropping systems. The development of such integrated digital agriculture systems will ultimately require collaboration among researchers, technology developers, and agricultural stakeholders to ensure that emerging Agriculture 5.0 technologies translate into practical and scalable solutions for growers. In addition, future progress will depend on the miniaturization, portability, and cost reduction in sensing systems so that maturity assessment tools become more accessible to growers with limited resources. Low-cost hand-held, mobile, or simplified field-deployable sensing options may be especially important for improving adoption at the grassroots level.

## 8. Conclusions

Peanut maturity estimation remains a cornerstone of successful and profitable peanut production, yet it continues to pose major challenges due to the crop’s indeterminate growth pattern, environmental variability, and reliance on subjective, labor-intensive traditional methods. While conventional approaches have provided valuable insights, their tedious nature, limited spatial applicability, and dependency on manual interpretation underscore the pressing need for scalable, objective, and technology-driven alternatives. Non-invasive techniques ranging from proximal sensing to Earth observation-based remote sensing have emerged as promising tools to address these limitations. The integration of artificial intelligence (AI) within these systems has further advanced the automation, precision, and adaptability of peanut maturity estimation. Despite these advances, current methods often lack the comprehensive incorporation of agroclimatic, phenological, and cultivar-specific factors, which restrict their robustness and transferability across diverse production environments. Moreover, the translation of maturity estimates into actionable harvest decisions remains an unresolved challenge that must be addressed for these technologies to achieve practical relevance at the farm level. Based on the reviewed literature, no single algorithm can be universally identified as optimal for peanut maturity monitoring. However, multivariate approaches such as PLSR and PLSDA have shown robustness for high-dimensional spectral data and VIs, while ML models including artificial neural networks (ANNs) and random forest (RF) demonstrate improved performance when integrating multi-source datasets. The suitability of a given approach depends on data availability, sensing platform, cultivar type, and application scale, with simpler models offering interpretability and more complex models enabling enhanced predictive capability under variable field conditions. Future efforts should focus on developing end-to-end digital maturity estimation pipelines that integrate spatial big-data analytics, centralized data management, and intuitive graphical user interfaces, all supported by cyber-secured cloud computing frameworks. Such infrastructure would enable seamless fusion of diverse data streams, including spectral, environmental, and cultivar-specific information, thereby improving predictive accuracy and operational efficiency. Achieving this vision will demand extensive ground-truth data collection across multiple seasons, varieties, and agroclimatic regions to ensure model generalizability and reliability. Ultimately, achieving a robust, field-deployable digital infrastructure for peanut maturity assessment will hinge on interdisciplinary and cross-sectoral collaboration among agronomists, data scientists, growers, crop consultants, and technology providers. These collaborations will not only accelerate innovation but also enhance economic sustainability, environmental resilience, and overall quality of life for peanut producers worldwide. By bridging traditional agronomic expertise with modern digital intelligence, the future of peanut maturity estimation stands poised to become more precise, automated, operationally relevant, and impactful than ever before.

## Figures and Tables

**Table 1:** Description of peanut growth stages, including the number of days from planting required to achieve each stage.

Phenological Stage †	Stage Name	Days After Planting	Description
V_E_	Emergence	3–7	Cotyledons near the soil surface with the seeding showing some part of the visible plant
V_0_			Cotyledons are flat and open at or below the soil surface.
V_1_	First tetrafoliate leaf		First tetrafoliate leaf is unfolded, and its leaflets are flat.
V_N_	Nth tetrafoliate leaf		N developed nodes on the main axis with tetrafoliate leaves
R_1_	Beginning bloom	30–45	One open flower at any node on the plant
R_2_	Beginning peg	55	One elongated peg (gynophore)
R_3_	Beginning pod	70	One peg in the soil with a turned swollen ovary is at least twice the width of the peg.
R_4_	Full pod	75	One fully expanded pod, to dimensions characteristic of the cultivar
R_5_	Beginning seed	80	One fully expanded pod in which seed cotyledon growth is visible when the fruit is cut in a cross-section with a razor blade (past the liquid endosperm phase).
R_6_	Full seed	90	One pod with a cavity apparently filled by the seed when fresh
R_7_	Beginning maturity	130	One pod shows visible natural coloration or blotching of the inner pericarp or testa.
R_8_	Harvest maturity	150–160	Two-thirds to three-fourths of all developed pods have testa or pericarp coloration. Fraction is cultivar-dependent and lower for Virginia type.
R_9_	Over-mature pod	161–170	One undamaged pod showing orange-tan coloration of the testa and/or natural peg deterioration.

† For populations, V stages can be averaged if desired. Reproductive stages should not be averaged. An R stage should remain unchanged until the date when 50% of the plants in the sample demonstrate the desired trait of the next R stage. The timing of a reproductive stage for a given plant is set by the first occurrence of the specific trait on the plant without regard to its position on the plant (adapted from Boote, [9]).

**Table 2:** Comparative overview of traditional peanut maturity assessment methods.

Method	Destructive/Non-Destructive	Labor Intensity	Field Applicability	Cost/ Equipment Needed	Strengths	Limitations
Shellout (interior hull color)	Destructive	High	Low to moderate	Low to moderate (higher if a color meter used)	Simple, historically established, can be improved with color-measuring devices	Labor-intensive; subjective visual classification; limited sampling accuracy and poor field-scale representativeness
Methanolic Extract (ME)	Destructive	High	Low	Moderate to high; requires methanol, blending setup, and spectrophotometer	Objective, quantitative, repeatable, economically informative harvest-window indicator	Requires many field samples; laborious; chemical handling and instrument dependence make grower adoption impractical
Arginine maturity Index (AMI)	Destructive	High	Low	High; requires kernel processing and automated colorimetric/laboratory analysis	Objective and automated; linked with yield and market-grade quality	Cultivar- and region-sensitive; affected by extreme weather; laborious sample collection; impractical for routine grower use
Seed Hull Matrix Index (SHMI: FMI/DMI)	Destructive	High	Low to moderate	Moderate	Quantitative seed-to-hull maturity indicator; associated with yield and quality	Time-consuming; requires repeated sampling and fresh/dry weight measurements; subject to sampling-location bias; at least about a week may be needed for full evaluation of multiple samples
Williams and Drexler (Hull Scrape) with MPB	Destructive	Moderate to high	Moderate	Low to moderate; pod blasting setup is needed	Widely used, practical, visually intuitive, directly linked to digging decisions; PMI can quantify maturity and is associated with yield, grade, and market value	Labor-intensive, pod preparation; subjective color interpretation; undefined field sampling protocols; repeated campaigns may be needed for field representativeness
Thermal Time/AGDD	Non-Destructive (Indirect)	Low to moderate	Moderate to high	Moderate to high; requires cultivar specific calibration	Objective, environmental maturity proxy; useful for growth-stage prediction, irrigation scheduling, harvest optimization, and genotype comparison	Sensitive to water deficit and other environmental modifiers; photoperiod/soil effects may need inclusion; cultivar-specific thresholds needed; weak for within-field spatial variability unless multiple sensors are installed

**Table 3:** Vegetation indices derived from remote sensing data and used for assessing peanut maturity.

Vegetation Indices	Acronyms	Formulas	References
Normalized Difference Vegetation Index	NDVI	$\frac{\left(NIR-RED\right)}{\left(NIR+RED\right)}$	[88]
Green Normalized Difference Vegetation Index	GNDVI	$\frac{\left(NIR-GREEN\right)}{\left(NIR+GREEN\right)}$	[89]
Non-Linear Index	NLI	$\frac{\left({NIR}^{2}-RED\right)}{\left({NIR}^{2}+RED\right)}$	[90]
Modified Non-Linear Index	MNLI	$\frac{\left({NIR}^{2}-RED\right)(1+L)}{\left({NIR}^{2}+RED+L\right)},$ where L = 0.5	[91]
Normalized Difference Red Edge Index	NDRE	$\frac{\left(NIR-RE\right)}{\left(NIR+RE\right)}$	[89]
Simple Ratio Index	SR	$\frac{NIR}{RED}$	[92]
Modified Non-Linear Red Edge Index	MNLIRE	$\frac{\left({NIR}^{2}-RE\right)(1+L)}{\left({NIR}^{2}+RE+L\right)},$ where L = 0.5	[85]
Simple Ratio Red Edge Index	SRRE	$\frac{NIR}{RE}$	[85]
Chlorophyll Red Edge Index	ChlRE	$\frac{NIR}{RE}-1$	[93]
Modified Simple Ratio Red Edge Index	MSRRE	$\frac{(NIR/RE)-1}{\sqrt{(NIR/RE)+1}}$	[94]
Non-Linear Red Edge Index	NLIRE	$\frac{\left({NIR}^{2}-RE\right)}{\left({NIR}^{2}+RE\right)}$	[85]

Note: In the reviewed peanut maturity literature, these vegetation indices (VIs) are primarily used for indirect maturity estimation from aboveground canopy spectral response. Platform compatibility is predominant in UAS, satellite, and occupied-aircraft multispectral systems, with red-edge indices requiring red-edge-capable sensors. Because the current peanut maturity literature remains limited, index-specific stage sensitivity and representative performance are not yet established uniformly across studies.

**Table 4:** Comparison of artificial intelligence approaches utilized for peanut maturity estimation with their operational accuracy, limitations and advantages.

Algorithm/Approach	Typical Models	Input Data	Accuracy	Computational Complexity	Advantages	Applicable Scenarios	Practical Challenges	Refs.
PLSR (Pod-based)	PLSR	Spectral (Vis–NIR reflectance)	R^2^ = 0.94	Low–moderate	Effective for high-dimensional spectral data; robust to collinearity	Laboratory or sample-scale maturity assessment	Labor-intensive; limited scalability	[73]
PLSR (Canopy-based)	PLSR	Multispectral imagery (VIs)	Good performance after 60 DAP	Low–moderate	Simple implementation; suitable for spectral data	Early canopy-based maturity estimation	Limited non-linear modeling capability	[86]
ANN (Canopy-based ML)	MLP, RBF	Multispectral (UAS/satellite) + climate (GDD)	R^2^ = 0.87–0.91; RMSE = 0.062; MAE = 0.05	High	Captures non-linear relationships; integrates multi-source data	Field-scale maturity estimation	Requires large datasets; risk of overfitting	[16,87]
Deep Learning (Canopy-based)	MLP, RBF, DL frameworks	Multi-source (imagery + climate + spectral)	R^2^ = 0.90–0.93; cross-site R^2^ = 0.59–0.64	High–very high	Learns hierarchical features; reduces feature engineering	Cross-field and large-scale prediction	Limited transferability; high data demand	[102,104]
DL (Pod/Image-based)	CNN, image processing + DL	RGB, HSV, LAB imagery	92.5% for mature class, 66.5% stage classification	Moderate–high	Automated feature extraction; objective classification	Controlled pod maturity classification	Requires preprocessing; labor-intensive sampling	[75]
Hyperspectral DL Models	ADNRNN	Hyperspectral seed data	~89% accuracy	Very high	Captures complex spectral patterns	Automated seed maturity grading	High computational demand; limited validation	[105]
Multi-target ML Frameworks	Multi-output regression	Satellite spectral + agronomic	>90%	Moderate–high	Simultaneous prediction (biomass + maturity)	Integrated crop monitoring	Limited multi-environment validation	[102]
Phenology-Aware Models	RF, SVR, ANN, DL	Multi-temporal spectral + climate	R^2^ up to 0.82	Moderate–high	Captures growth dynamics; improves robustness	In-season maturity tracking	Requires time-series data	[101]

## Data Availability

This article is a review of published literature. No new data were created or analyzed in this study. All relevant information is contained within the manuscript and its cited references.
